# Genetics of heifer reproductive traits in Japanese Black cattle

**DOI:** 10.5713/ajas.19.0118

**Published:** 2019-05-28

**Authors:** Asep Setiaji, Takuro Oikawa

**Affiliations:** 1 United Graduate School of Agricultural Sciences, Kagoshima University, Korimoto, Kagoshima 890-0065, Japan; 2 Faculty of Agriculture, University of the Ryukyus, Nishihara, Okinawa 903-0213 Japan; 3 Department of Animal Science, Faculty of Animal and Agricultural Sciences, Diponegoro University, Semarang, 50275 Central Java, Indonesia

**Keywords:** Reproductive Traits, Japanese Black Heifer, Heritability, Genetic Correlation

## Abstract

**Objective:**

The objective of this study was to identify environmental factors strongly associated with and to estimate genetic parameters of reproductive traits in Japanese Black heifers.

**Methods:**

Data included reproduction records of Japanese Black heifers born between 2004 and 2014. First service non-return rate (NRR) to 56 days from first to successful insemination (FS), number of services per conception (IN), age at first calving (AFC) and gestation length were analyzed with the use of the general linear model. Genetic parameters were estimated with the use of the univariate animal model of the residual maximum likelihood.

**Results:**

Averages of reproductive traits over eleven years were assessed, and the effects of farm, year, month, artificial insemination technician and interaction of farm×year on the traits were determined. Estimated heritability of FS was very low and that of AFC was higher than that of the other traits. A close genetic relation was observed among NRR, IN, and FS; however, their heritabilities were very low. AFC shows favorable genetic correlation with IN and FS.

**Conclusion:**

Low heritabilities of most reproductive traits in Japanese Black heifers are strongly influenced by farm management practices, and that large residual variances make genetic evaluation difficult. Among the reproductive traits, AFC is potentially more useful for genetic improvement of heifer reproductive traits because it has high heritability and favorable genetic correlations with IN and FS.

## INTRODUCTION

Variation in the reproductive performance of cattle is dependent on genetic and environmental factors. The observed performance of a trait in cattle is the result of heredity and the effects of the environment where the animal is raised. Genetic improvement of female reproductive traits is currently attracting great interest in genetic evaluation of beef cattle because of the influence of such traits on the productivity of calves. Difficulties in measuring these traits and the low genetic variability result in slow response to selection [1,2]. Reproductive traits in heifers can be measured at a relatively early stage of their reproductive life; thus, the inclusion of heifer reproductive traits in genetic evaluation programs is potentially effective.

Most reproductive traits in heifers are of low heritabilities in dairy cattle [3–5] and in beef cattle [6–8]. The number of reports on reproductive traits of Japanese Black heifers is relatively small. Low heritabilities of reproductive traits is attributed to non-genetic effects, especially farm management practices that largely affect the reproductive performance of heifers [9]. Selection of reproductive traits in heifers based on genetic parameters may potentially solve reproduction problems in the raising of beef calves.

Reproductive traits of heifers and cows need to be evaluated separately, although reproductive traits in heifers genetically correlate strongly with reproductive traits in subsequent parities [10]. Therefore, designing breeding programs at an early stage, necessitates the selection of reproductive traits of as high heritabilities as possible and of favorable genetic correlations with other reproductive traits. Here, the study is directed at identifying non-genetic factors strongly associated with reproductive traits and to estimate genetic parameters of reproductive traits in Japanese Black heifers.

## MATERIALS AND METHODS

Reproduction records of Japanese Black heifers were obtained from the Artificial Insemination Center of Northern Okinawa: data comprised artificial insemination and calving events, five reproductive traits from artificial inseminations and calving from a registry book. The traits studied were first service non-return rate (NRR) to 56 days coded one (1), when no subsequent insemination occurred until 56 days after the first insemination, and zero (0) otherwise; days from first to successful insemination (FS) being the number of days between the first insemination and the successful insemination that resulted in conception; number of services per conception (IN); age at first calving (AFC) defined as the difference between the heifer’s date of birth and the date of birth of her first calf, and gestation length (GL) being the interval in days from the last insemination to the subsequent calving.

### Data set

A data set included reproduction records of heifers born between 2004 and 2014, first insemination between 2005 and 2015, less than 10 services and farms with a minimum of five records. Heifers with incomplete records, embryo transfer donor or recipient, and bearing twin calves were excluded from the data set. Edited records of 2,215 heifers under NRR and 2,047 heifers under FS, IN, AFC, and GL were analyzed. To evaluate the effects of age at first insemination, heifers were classified into three age groups: <16, 16 to 19, and >19 month [3]. Eight artificial insemination (AI) technicians carried out the artificial insemination procedure on all the heifers from the 164 farms. The number of animals in the pedigree was 15,600.

### Statistical analysis

The general linear model (GLM) procedure of Statistical Analysis System 9.3 (SAS) software [11] was used to test the significance of reproductive traits. The linear model for NRR, FS, IN, and GL was as follows:

$$y_{ijklmop}=F_{i}+Y_{j}+M_{k}+T_{l}+A_{m}+F_{i}\times Y_{j}+s_{o}+e_{ijklmop}$$

where *y_ijklmop_* is the observation of NRR, FS, IN, and GL, *F_i_* the ith fixed effect of farm, *Y_j_* the jth fixed effect of year of insemination, *M_k_* the kth fixed effect of month of insemination, *T_l_* the lth fixed effect of AI technicians, *A_m_* the mth fixed effect of age at first insemination, *s_o_* the oth random effect of sire, and *e_ijklmop_* the random residual of *y_ijklmop_*.

The following model was used for AFC:

$$y_{ijklop}=F_{i}+Y_{j}+M_{k}+T_{l}+F_{i}\times Y_{j}+s_{o}+e_{ijklop},$$

where *y_ijklop_* is the observation of AFC. *F_i_*, *T_l_*, and *s_o_* are as described in the previous model where two effects were defined differently, *Y_j_* the jth fixed effect of year of birth, *M_k_* the kth fixed effect of month of birth and *e_ijklop_* the random residual of *y_ijklop_*. The GLM procedure of SAS 9.3 software [11] was used to estimate the least square mean (LSM) of the year effect. The univariate animal model by residual maximum likelihood of the Asreml software [12] was used to estimate the genetic parameters. The linear model for the analysis was as follows:

y=Xb+Gu+e,

where, **y** = a vector of reproductive traits, **b** = a vector of fixed effect, **X** = an incidence matrix for the fixed effects, **u** = a vector of random genetic additive effect, **Z** = an incidence matrix for the random effect and **e** = a vector of random residuals.

The expectations for **y**, **u**, and **e** are

$$E\;\left[\begin{array}{c} y\\ u\\ e \end{array}\right]=\left[\begin{array}{c} \mathrm{Xb}\\ 0\\ 0 \end{array}\right].$$

The variance-covariance structure of random effects was

$$V\;\left[\begin{array}{c} u\\ e \end{array}\right]=\left[\begin{array}{cc} A\sigma_{a}^{2} & 0\\ 0 & I\sigma_{e}^{2} \end{array}\right],$$

where *A* is the numerator relation matrix, *I* the identity matrix, $\sigma_{a}^{2}$ and $\sigma_{e}^{2}$ are the additive genetic variance and residual variance, respectively.

## RESULTS

The averages of the reproductive traits over eleven years were 67%, 1.76, 36.33 days, 25.8 months and 288.22 days for NRR, IN, FS, AFC, and GL, respectively (Table 1). Farm effect was significant in all traits, year effect was significant in NRR, FS, GL, and AFC, and month effect was significant in only GL. The effect of AI technician was significant in FS and AFC, whereas age at first insemination was not significant in any of the traits; furthermore, the interaction of farm×year effect was significant in FS, AFC and GL (Table 2).

The highest LSM for NRR was observed in 2010, and for IN and FS in 2005. The longest GL was observed in 2010. Heifers born in 2010 showed the highest LSM for AFC (Table 3). Estimated heritability of FS was the lowest, and that of AFC was higher than of the other traits (Table 4).

The highest and the lowest genetic correlations were between FS and NRR and between FS and GL, respectively, in absolute values. The AFC showed moderate genetic correlation with NRR and GL, but high with IN and FS. The phenotypic correlation between NRR and GL was the lowest, whereas that between FS and AFC was the highest (Table 5).

## DISCUSSION

In the present study, NRR after the first service was slightly lower (67%) than in published results: 74% in Canadian Holstein heifers [13], and 73.9% in Holstein Friesian heifers in the Netherlands [14]. FS of Japanese Black heifers in Okinawa was 36.33 days and slightly longer than the average reported for the Rubia Gallega breed (30.60 days) [15]. The IN of Japanese Black cows has been 1.41 [16], whereas it was lower than the average in the present study; on the other hand, GL was consistent with their average (288 days). In this study, AFC was 25.8 months, being longer than 24.4, 25.1, and 24.98 months, as reported [17–19], and records in Okinawa prefecture between 1990 and 2009 have shown AFC at 26.93 months, being longer than in the present study [20].

In the present study, analysis of variance showed that farm had a significant effect on all reproductive traits of Japanese Black heifers in Okinawa, indicating that different management practices on farms influenced heifer reproductive traits significantly. Animal management standards on farms has a significant effect on the number of services to Holstein heifers [21]. Better reproductive performance of cows on large farms relative to those on small farms is attributable to high standard equipment and operating procedures [22]. In the present study, the year of insemination had a significant effect on NRR, FS and GL, and the year of birth had a significant effect on AFC. A similar study on the Simmental breed, has shown that the year effect is strong on female reproductive traits, the effect of the AI technician is significant on FS and AFC, but age at first insemination is not significant in any of the traits [9]. By contrast, reproductive traits in dairy heifers are influenced by age at first insemination: heifers inseminated at an older age for the first time have a slightly higher rate of success at first insemination and, as a result, require a smaller number of services [13]. Furthermore, environmental variations, employment of an estrus detector and the expertise of the AI technician may play major roles in a successful first insemination [23].

A previous study on Holstein heifers has shown that genetic variance in binary and count traits are lower than those in interval traits [10,24]. The linear statistical model is appropriate and common for genetic analysis of interval traits; however, it is not optimal for genetic analysis of binary traits, the nature of which violates assumptions of normality under which the linear model is valid [25]. On the other hand, the threshold model has proved to be appropriate for evaluating binary traits, and heritabilities obtained with its use has usually been higher than that obtained with the linear model for the same trait [13]. Nonetheless, a disadvantage of the threshold model is that it produces biased estimates for variances of random effects when categorical traits are analyzed together with interval traits for estimating variance components [26,27]. In the present study, five reproductive traits were analyzed, only one of which was binary. Genetic correlation among reproductive traits is difficult to estimate when pairs of trait are analyzed under different models; therefore, the linear model was used to estimate genetic parameters in this study.

In this study, heritability of NRR (0.027) was slightly higher than the estimate (0.012) for dairy heifers [3] with the use of a linear model, whereas it was 0.019 for IN and 0.011 for FS, which were in agreement with previous studies on dairy heifers, ranging between 0.013 and 0.026 and between 0.013 and 0.017 for IN and FS, respectively [3,5,10]. Furthermore, in the present study, the estimated heritability of GL (0.081) was consistent with that in in Japanese Black cows (0.080) [28], whereas it is lower (0.037) in the Rubia Gallega breed [15]. In our study, AFC was the reproductive trait that demonstrated the highest heritability (0.180), which was consistent with (0.215) in Japanese Black cattle [18], higher heritability (0.28) in Angus heifers [8], and lower heritability (0.09) in the Tabapuã beef breed [29].

In the present study, genetic correlations among pairs of the traits varied substantially: NRR showed negative and moderate genetic correlations with IN (−0.636) and GL (−0.569). Genetic correlation between NRR and IN was slightly lower to those estimated in Chinese Holstein heifers (−0.87) [10]. NRR showed negative and high correlation with FS (−0.913), similar to estimated value (−0.780) in Canadian Holstein by Jamrozik et al [13], whereas the genetic correlation for NRR and GL was low (0.25) in their study. The genetic correlation between FS and IN was favorably high (0.874) in the present study (Table 5), but slightly lower than a previous estimate of over 0.90 in dairy cows [10,13,30]. These high correlations seem to be attributable to the strong correlation between IN and FS. The genetic correlations between GL and IN and between GL and FS were low in the present study, but consistent with those in Rubia Gallega cows (−0.138) and (0.106), respectively [15]. IN and FS were recorded before conception, and GL indicates the capability of heifers to maintain pregnancy to calving day. Thus, the low genetic correlation indicates that these two traits (IN and FS) are genetically unrelated to GL and may be influenced by different groups of genes. Consequently, selection of one of the traits would have little effect on the other.

IN and FS showed high genetic correlations with AFC, whereas genetic correlations between AFC and NRR, and AFC and GL were moderate in absolute values. High genetic correlation seems to be due to the pleiotropic effect of genes, where a group of genes may simultaneously have a common genetic effect on these three traits (IN, FS, and AFC). Heifers that calve early tend to have fewer IN and shorter FS. Therefore, AFC is considered a key trait in the reproductive cycle of and as an indicator of sexual precocity in female cattle. Thus, AFC is potentially useful in genetic evaluation of heifer reproductive traits. Selection of AFC anticipates the achievement of higher genetic development of reproductive traits because of high heritability and favorable genetic correlations with IN and FS. The AFC is genetically correlated with calving interval in Japanese Black cows [18]. Heifers that conceive and drop the first calf early are expected to require a small number of inseminations and to undergo a short calving interval. Improvement of AFC in heifers plays an important role in increasing reproductive performance in subsequent parities.

## CONCLUSION

The present study suggests that low heritabilities of the most reproductive traits in Japanese Black heifers is strongly influenced by farm management practices, and that large residual variances make genetic evaluation difficult. The NRR, IN, and FS demonstrated close genetic correlation with one another, although their heritabilities were very low. Among the reproductive traits, AFC is potentially more useful for genetic improvement of heifer reproductive traits because it has high heritability and favorable genetic correlations with IN and FS.

## Figures and Tables

**Table 1:** Parameters of heifer reproductive traits

Traits	No. of records	Mean	SD	Minimum	Maximum
NRR	2,215	0.67	0.47	0	1
IN	2,047	1.76	1.08	1	10
FS (days)	2,047	36.33	69.88	0	363
AFC (months)	2,047	25.8	5.39	19.67	43.27
GL (days)	2,047	288.22	4.94	263	303

SD, standard deviation; NRR, non-return rate; IN, number of inseminations; FS, interval from first to successful insemination; AFC, age at first calving; GL, gestation length.

**Table 2:** Significance of factors affecting heifer reproductive traits

Source	df	Traits									

		NRR		IN		FS (days)		AFC (months)		GL (days)	
				
		F-value	p-value	F-value	p-value	F-value	p-value	F-value	p-value	F-value	p-value
Farm	163	1.27	0.0151	1.48	0.0002	1.81	<0.0001	3.07	<0.0001	4.27	<0.0001
Year	10	2.73	0.0016	1.5	0.1259	2.46	0.0048	3.16	0.0002	3.01	0.0005
Farm×year	636	1.04	0.2632	0.95	0.7883	1.33	<0.0001	1.38	<0.0001	1.43	<0.0001
Month	11	0.96	0.4796	1.13	0.3319	0.93	0.514	0.85	0.5848	3.64	<0.0001
Technician	7	1.19	0.3124	1.43	0.2105	2.31	0.0423	2.95	0.0117	0.38	0.8635
Age	2	0.45	0.6399	1.73	0.1783	2.26	0.1049	-	-	0.98	0.3767

NRR, non-return rate; IN, number of inseminations; FS, interval from first to successful insemination; AFC, age at first calving; GL, gestation length.

**Table 3:** Yearly trend of least square mean (±standard error) of heifer reproductive traits

Year1)	Traits				

	NRR	IN	FS	AFC	GL
2004	-	-	-	26.28±0.45	-
2005	0.64±0.04	1.99±0.11	100.83±10.76	25.55±0.43	287.20±0.42
2006	0.69±0.04	1.85±0.10	53.58±9.92	26.03±0.35	287.00±0.39
2007	0.62±0.03	1.83±0.09	66.48±8.80	26.16±0.35	288.45±0.34
2008	0.72±0.03	1.77±0.08	73.08±8.07	26.05±0.34	288.59±0.31
2009	0.68±0.03	1.71±0.08	49.05±7.91	26.24±0.33	288.80±0.31
2010	0.74±0.03	1.79±0.08	61.35±7.95	26.43±0.38	289.54±0.31
2011	0.65±0.03	1.98±0.09	73.05±8.71	25.26±0.44	288.93±0.34
2012	0.73±0.04	1.75±0.10	64.13±9.51	25.50±0.44	288.50±0.37
2013	0.70±0.04	1.77±0.11	52.77±10.46	25.36±0.39	287.94±0.41
2014	0.60±0.03	1.85±0.10	42.11±9.55	24.10±0.42	286.90±0.37
2015	0.67±0.03	1.63±0.10	31.26±9.40	-	287.57±0.37

NRR, non-return rate; IN, number of inseminations; FS, interval from first to successful insemination; AFC, age at first calving; GL, gestation length.

1) Year of insemination for NRR, IN, FS, and GL (2005–2015); year of birth for AFC (2004–2014).

**Table 4:** Genetic parameters (±standard error) of heifer reproductive traits

Traits	Genetic variance	Error variance	Heritability
NRR	6.05×10^−3^±0.005	0.21±0.007	0.027±0.024
IN	3.07×10^−2^±0.093	1.58±0.068	0.019±0.023
FS	121.85±0.332	1026±0.532	0.011±0.018
AFC	5.26±0.061	24.17±0.012	0.180±0.028
GL	1.42±0.016	15.91±0.094	0.081±0.024

NRR, non-return rate; IN, number of inseminations; FS, interval from first to successful insemination; AFC, age at first calving; GL, gestation length.

**Table 5:** Genetic correlation above diagonal and phenotypic correlation below diagonal (±standard error) among reproductive traits

Traits	NRR	IN	FS	AFC	GL
NRR	-	−0.636±0.031	−0.913±0.028	−0.351±0.009	−0.569±0.047
IN	−0.551±0.016	-	0.874±0.025	0.625±0.048	0.226±0.026
FS	−0.162±0.023	0.708±0.011	-	0.798±0.034	0.183±0.008
AFC	−0.057±0.022	0.511±0.018	0.881±0.008	-	0.299±0.021
GL	−0.037±0.024	0.080±0.022	0.043±0.025	0.129±0.024	-

NRR, non-return rate; IN, number of inseminations; FS, interval from first to successful insemination; AFC, age at first calving; GL, gestation length.
